# Methylphenidate for treating fatigue in palliative cancer care – effect and side effects in real-world data from a palliative care unit

**DOI:** 10.2340/1651-226X.2024.24156

**Published:** 2024-02-13

**Authors:** Agneta Almerud, Gabriella Frisk, Caritha Klasson, Linda Björkhem-Bergman

**Affiliations:** a ASIH Stockholm Södra, Palliative Home Care and Specialized Palliative Ward, Bergtallsvägen 12, SE-125 59 Älvsjö, Sweden; b Department of Neurobiology, Care Sciences and Society (NVS), Division of Clinical Geriatrics, Karolinska Institutet, Blickagången 16, Neo floor 7, SE-141 83, Huddinge, Sweden; c Stockholms Sjukhem, Palliative Medicine, Mariebergsgatan 22, SE-112 19, Stockholm, Sweden

**Keywords:** Fatigue, palliative care, methylphenidate, dose-response, adverse effects

## Abstract

**Background:**

Methylphenidate can be used for the treatment of cancer-related fatigue (CRF), although randomized controlled trials have shown conflicting results. The aim of this study was to use ‘real-world’ data to evaluate the effect and side effects of using methylphenidate in palliative cancer care with a focus on the late palliative phase and dose-response.

**Method:**

A retrospective review of medical records from a palliative care unit in Sweden was performed to evaluate the effect and adverse events (AEs) of using methylphenidate to treat CRF. Univariable and multivariable regression was performed and odds ratio (OR) calculated. Adjustments were made for sex, age, cancer type, dose and starting treatment <4 weeks before death.

**Results:**

Of the 2,419 screened patients, 112 had been treated with methylphenidate for CRF. The treatment was assessed as being effective in 51 patients (46%). Twenty-six patients (23%) experienced AEs that were generally mild, including anxiety, palpitations, and insomnia. Patients starting the treatment <4 weeks before death (*n* = 54) were less likely to have an effect from treatment compared to those starting earlier; adjusted OR 0.24 (95% CI 0.10–0.55). Doses of 20 mg and above were well-tolerated and had a higher frequency of effect in the crude data but not after adjustment for confounding factors.

**Conclusion:**

Methylphenidate is generally effective and well-tolerated for the treatment of CRF in palliative care. However, patients with a short life expectancy (<4 weeks) seem to benefit less from the treatment regardless of age, cancer type and dose.

## Introduction

Cancer-related fatigue (CRF) is one of the most distressing symptoms experienced by patients with cancer receiving palliative care and which substantially affects their quality of life (QoL) [1–5]. Fatigue is often a complex symptom where several different factors may contribute, both physical and psychological. A tailored treatment is, therefore, often needed for each patient, which might include both pharmacological and non-pharmacological treatment options [6–10].

In a recent systematic review, we evaluated the evidence for pharmacological interventions for treating CRF in palliative cancer care, comprising randomized controlled trials (RCTs) of different drugs, including psychostimulants [6]. We concluded that the evidence for using pharmacological treatments for CRF is still weak, but that methylphenidate might be an option to consider. This is also in line with recommendations from the United States National Comprehensive Cancer Network (NCCN), which states that methylphenidate can be considered in the treatment of fatigue in patients with cancer at the end of life [11]. The European Society for Medical Oncology (ESMO) is more hesitant regarding the use of methylphenidate [12].

Regarding psychostimulants, 11 RCTs have been performed on CRF, 7 with methylphenidate [13–19], 3 with modafinil [20–22] and 1 with dexamphetamine [23].

The results reveal a significant effect that is superior to placebo in only three of the seven trials of methylphenidate [17–19] and in none of the trials of modafinil or dexamphetamine. Interestingly, 8 of the ‘negative’ studies showed a significant effect in both the placebo and intervention arms [13–16, 20–23]. All the RCTs showed that the use of methylphenidate was safe and had either no or mild adverse events (AEs). In addition, one open-label study has been performed on the use of methylphenidate for depression in patients with advanced cancer, also showing positive effects on both depression and fatigue [24].

Notably, the doses and the settings used in the RCTs differed markedly between the studies. The doses ranged from 5 to 30 mg/day and the treatment length from 3 days to 10 weeks. Moreover, the majority of the RCTs were performed in patients who were early in the disease trajectory, including both patients receiving curative and palliative oncological treatments. Only three of the RCTs included patients in a late palliative phase of their disease, that is during the last weeks and months of life [14, 16, 17]. There is, therefore, still limited knowledge about whether methylphenidate has an effect in late-stage cancer disease, especially during the last weeks of life.

According to the local guidelines in our unit, the starting dose of methylphenidate is often 5–10 mg in the morning which is then titrated up to 20 mg or more after a few days [25]. If a two-dose procedure is used, methylphenidate is administrated as one dose in the morning and one dose at lunchtime [25]. According to our clinical experience, daily doses of 20 mg are often required to achieve a substantial effect in patients with advanced cancer and severe fatigue.

The most common AEs of methylphenidate are insomnia, anxiety, decreased appetite, and weight loss [13–19]. An increase in blood pressure and palpitations are also common [26]. Thus, cardiovascular disease is a relative contraindication for treatment with methylphenidate. Patients suffering from schizophrenia, psychotic disorders, or severe bipolar disorders should not be treated with methylphenidate due to the risk of a negative impact on their psychiatric disease [27–29]. In addition, patients with dementia and delirium are generally not treated with methylphenidate in palliative care.

We have used methylphenidate for several years to treat CRF in palliative care patients in our Palliative Home Care and Specialized Palliative Ward in Stockholm, ASIH Stockholm South. Our experience is that it is safe, has mild AEs, but that the effect varies from very good to no effect at all.

In this study, we aim to describe the effect and side effects of methylphenidate in data obtained from a ‘real-world’ cohort in our palliative care unit and to compare these with the results from RCTs [13–19]. In addition, we wanted to test the hypothesis that methylphenidate could be effective in the treatment of fatigue even in a late palliative phase, that is, the last weeks in life, and that doses of at least 20 mg are often needed to achieve an effect.

## Method

### Study design

This was a retrospective, observational study where medical records from all patients admitted to the Palliative Home Care Unit and Specialized Palliative Ward in Stockholm ‘ASIH Stockholm South’ from 01 January 2016 to 31 December 2018 were reviewed. The study was approved by the Swedish Ethical Review Board, Dnr 2018/1798-31. All patients included were deceased at the time of the retrospective review; no informed consent was therefore obtained.

### Study setting

The Palliative Home Care Unit ‘ASIH Stockholm South’ admits both oncological and non-oncological patients, at different stages of their disease trajectory, who are in need of supportive and/or palliative care at home. Geographically, the unit admits patients from the southern part of Region Stockholm, which has approximately 800,000 inhabitants and covers mostly low- and middle-income areas. The average length of care is 3 to 4 months. The unit also has a 16-bed palliative in-patient ward providing end-of-life care, where the average care time is 1 to 2 weeks. More details about the unit can be found elsewhere [30].

Symptom assessment is regularly monitored in most patients admitted to the unit using the Edmonton Symptom Assessment System (ESAS). The results are logged in the medical records. The ESAS includes a question about tiredness/fatigue with a score from 0 to 10, where 0 is ‘no fatigue’ and 10 is ‘maximum fatigue’.

### Participants

Patients from both the home care unit and the in-patient ward were included in the study. The inclusion criteria were treatment with methylphenidate for at least 3 days where the major indication for treatment was fatigue/tiredness. The reason for choosing 3 days was that this was the shortest study period used in the RCTs of methylphenidate [16]. In addition, only patients with advanced cancer and in a palliative phase were included. Patients treated with methylphenidate due to attention deficit hyperactivity disorder (ADHD) and those with non-cancer diseases were excluded. No central stimulants other than methylphenidate were prescribed.

For all patients fulfilling the inclusion criteria, data were extracted from the medical records regarding age, sex, type of cancer, dose/doses of methylphenidate, length of treatment, symptom assessment before, during, and after treatment (if available) using ESAS including the fatigue-score. The reason for termination of treatment was also recorded.

If the dose had been changed during the treatment, the mean dose over the treatment period was used.

In order to evaluate the effect when starting treatment during the last weeks of life, patients starting their treatment < 4 weeks or > 4 weeks before death were compared. In addition, subgroup analysis was performed between men and women, between patients taking doses of 5–15 mg/day compared to 20–50 mg/day, and between different cancer types.

### Variables

The main outcome was effectiveness of treatment categorized into ‘yes’ or ‘no’. The medical records of the patients meeting the inclusion criteria were independently reviewed by AA and GF to assess the effect. Both AA and GF are senior consultants at the unit and specialists in oncology and palliative medicine. The assessment of the effect was performed by reading the medical records noted just before and after starting treatment. Usually, the treatment was evaluated within 1 week and the effect recorded by the home care team or the staff on the palliative ward. Any change in ESAS score was also assessed when this was available. In addition, a search tool for screening all text in the medical record for the word ‘tiredness’ was used.

If the assessors had made differing evaluations regarding the effect, the case was discussed, and consensus was reached. The secondary outcome was AEs of the treatment, which was also retrieved from the medical records. In addition, the reason for termination of treatment was analyzed.

In the main analysis, patients were divided in two groups: those ‘starting treatment < 4 weeks before death’ and those ‘starting treatment > 4 weeks before death’.

Secondary analysis was performed comparing men and women, different cancer types, and comparing patients treated with low dose (5–15 mg/day) and high dose (20–50 mg/day) of methylphenidate.

### Study size

This was an observational descriptive cohort study where all patients who fulfilled the inclusion criteria during the study period of 3 years were included; no power calculation was therefore performed.

### Statistical methods

Descriptive statistics are presented as median and interquartile range (IQR) or n (number) and percentage (%). Differences between the groups were assessed using the Mann-Whitney test since most data did not show Gaussian distribution. Fisher’s exact test was used for categorical variables. Univariable followed by multivariable regression was performed and adjusted for sex, age, cancer type, and dose (categorized into low dose 5–15 mg/day or high dose 20–50 mg/day). Cancer types were categorized into three categories: gastro-intestinal (GI) cancers, potential hormone-dependent cancers (breast/prostate), and others. Age was a continuous variable in the model and all other variables were categorized: sex (female/male), cancer type (3 types). Statistical analysis was performed in GraphPad Prism, vs 9.0.

## Results

### Participants

A total of 2,419 medical records were screened, including all patients admitted to the unit between 2016 and 2018. Of these, 121 patients had been treated with methylphenidate. Two of these had been treated with methylphenidate due to ADHD and one due to fatigue related to amyotrophic lateral sclerosis (ALS). Six patients had only been treated for 1–2 days. These 9 patients were all excluded. In the final analysis, the study population comprised 112 patients with advanced cancer, 61 men and 51 women, who had been treated with methylphenidate and where fatigue was the main indication. No patient with dementia or delirium had been treated with methylphenidate. The demographic data describing the study cohort are presented in Table 1.

**Table 1:** Demographic data of the study cohort. Values show median and interquartile range for age and ESAS. Amount (n) and % are presented for the other variables.

	All (*n* = 112) (%)	Men (*n* = 61) (%)	Women (*n* = 51) (%)
Age (years)	69 (62–76)	69 (62–77)	70 (62–75)
Starting treatment > 4 weeks bf death	58 (52)	30 (49)	28 (55)
Starting treatment < 4 weeks bf death	54 (48)	31 (51)	23 (45)
ESAS fatigue at baseline* (score 0–10)	7 (4–8)	7 (4–7)	7 (4–8)
**Types of cancer**			
Prostate	15 (13)	15 (25)	0 (0)
Upper GI	11 (10)	6 (10)	5 (10)
Lower GI	12 (11)	7 (12)	5 (10)
Lung	13 (12)	7 (12)	6 (12)
Breast	10 (9)	0 (0)	10 (20)
Hematological	8 (7)	6 (10)	2 (4)
Gynecological	8 (7)	0 (0)	8 (16)
Pancreas	17 (15)	10 (16)	7 (14)
Head-Neck	4 (4)	1 (2)	3 (6)
Brain	2 (2)	2 (3)	0 (0)
Urological	3 (2)	1 (2)	2 (4)
Melanoma	3 (2)	2 (3)	1 (2)
Liver	2 (2)	2 (3)	0 (0)
Other	4 (4)	2 (3)	2 (4)

ESAS: Edmonton Symptom Assessment System; GI: gastro-intestinal.

* ESAS was available for only 69 patients.

After the first review, the concordance between the two assessors regarding the effect on fatigue was 96%. Five cases were discussed, and consensus was reached regarding effect ‘yes’ or ‘no’. The ESAS assessment was only available for 69 patients and only 16 patients had ESAS scores recorded both before and during treatment. We chose, therefore, to only present the ESAS score at baseline to obtain some idea about the assessed fatigue in the cohort.

Of the 112 patients, 54 had started their treatment < 4 weeks before death and 58 had started > 4 weeks before death.

### Doses and treatment length

**Figure 1:**
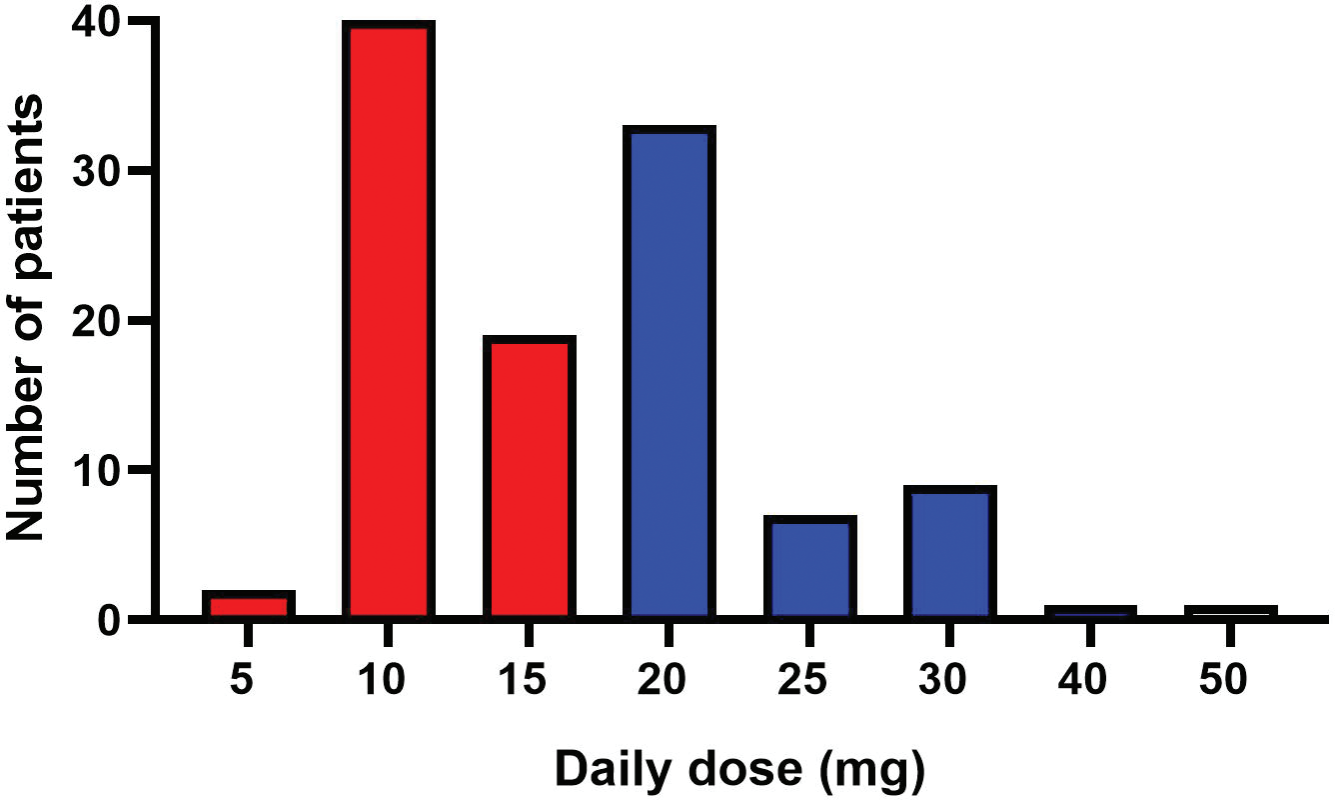
Frequency of methylphenidate doses in the study cohort, *n* = 112. In this study, 5–15 mg is considered ‘low dose’ (red color) and 20–50 mg/day ‘high dose’ (blue color).

The most common doses prescribed were 10 mg or 20 mg. The vast majority of the patients had been treated with methylphenidate as ‘instant release’ (92%). The dose was given either once in the morning (most common) or as 10 mg in the morning and 10 mg at midday (no later than 2 pm). The highest dose used was 50 mg. The doses in the study cohort are shown in Figure 1. Sixty-one patients had been treated with doses of 5–15 mg, defined as ‘low dose’ in the analysis, and 51 had been treated with 20–50 mg/day, defined as ‘high dose’. The dose was generally increased up to the target dose during the first days of treatment. However, in some cases the doses were adjusted (increased or decreased) after several weeks or even months. In those cases, the mean dose over the treatment period was used. Among the patients starting the treatment <4 weeks before death, the median dose was 10 mg and for those starting earlier than 4 weeks before death, the median dose was 15 mg (*p* < 0.01).

The effect of methylphenidate was generally instant, within hours of the dose being given. In some cases, it took a day or two before an effect could be recognized.

The median treatment length was 16 days, ranging from 3 days to more than 1 year. The most common reasons for termination of treatment were deterioration and death (59%). The lack of effect or decreased effect was the reason in 19% and AE was the reason in 13% of the cases.

### Effect of treatment

In the whole study cohort, 51 of 112 patients (46%) had received a positive effect from the treatment according to our assessment. A positive effect was more common among patients starting the treatment > 4 weeks before death (62%) compared to those starting < 4 weeks before death (28%) (*p* < 0.001). The proportion of patients in which the treatment had an effect was similar between men and women and between different age groups (data not shown). The proportion that had received an effect was also similar between different types of cancers, except for hematological malignancy where none of the 8 patients had received an effect. Moreover, methylphenidate seemed to have less effect when the fatigue was caused by infections or anemia and better effect when depression or opioid-induced fatigue was contributing to or causing the fatigue.

Patients taking doses of 5–15 mg/day were less likely to receive an effect than those taking 20–50 mg/day; 36% compared to 57% (*p* < 0.05).

### Adverse events

Among the 112 patients, 23% experienced some AEs, assessed as mild or moderate. The most common AEs were increased anxiety and insomnia. The number of AEs was similar in the patients starting the treatment <4 weeks before death and those starting earlier than 4 weeks before death, 20% compared to 26% (*p* = 0.66).

**Figure 2:**
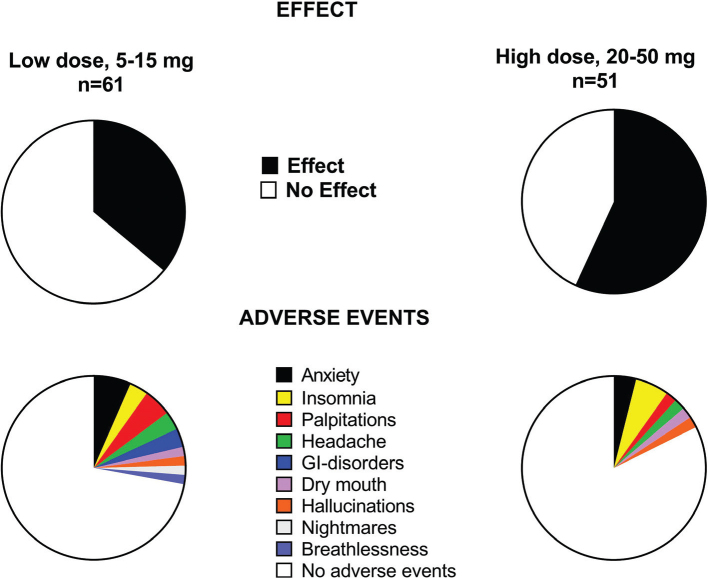
Effect and adverse events in 112 patients treated with low-dose and high-dose methylphenidate for cancer-related fatigue in palliative care.

The number of AEs was also similar between those taking a high dose of 20–50 mg/day compared to those taking a low dose of 5–15 mg/day; 28% compared to 18% (*p* = 0.26). The pattern of AEs and effect in relation to dose are shown in Figure 2.

### Univariable and multivariable regression model of effect

The odds ratio (OR) in patients starting the methylphenidate treatment <4 weeks before death was significantly lower than for those starting the treatment > 4 weeks before death; crude OR 0.18 (95% CI 0.07–0.40) and adjusted OR 0.24 (95% CI 0.10–0.55) (Table 2). Patients taking a low dose (5–15 mg/day) were less likely to receive an effect compared to those taking a high dose in the univariable model (Table 2). However, after adjustment for age, sex, cancer type and starting the treatment < 4 weeks before death, the differences between the group were no longer significant. Sex, age and cancer type did not affect the OR of effect (Table 2).

**Table 2:** Univariable and multivariable regression on the effect of methylphenidate for cancer-related fatigue in palliative care. Hormonal dependent cancers comprise breast and prostate cancers. Age was a continuous variable while all other variables were categorized.

Variable	*n*	Univariable analysis	Multivariable analysis
		OR (95% CI)	OR (95% CI)
**Start treatment** > 4 weeks bf death < 4 weeks bf death	58 54	Ref. 0.18 (0.07–0.40)***	Ref. 0.24 (0.10–0.55)***
**Dose** Low-dose (5–15 mg/day) High-dose (20–50 mg/day)	61 51	Ref. 2.34 (1.12–5.13)***	Ref. **1.92 (0.83–4.37)**
**Sex** Male Female	61 51	Ref. 1.29 (0.63–2.20)	Ref. 1.31 (0.57–3.03)
**Age** (range 29–95 year)	112	0.99 (0.96–1.02)	0.98 (0.95 – 1.01)
**Cancer types** Other types Hormone dependent GI-cancers	44 25 43	Ref. 1.14 (0.48–2.63) 0.84 (0.38–1.81)	Ref. 1.03 (0.35–3.08) 1.03 (0.41–2.64)

Abbreviations: OR: odds ratio; CI: confidence interval; Bf: before; GI: gastro-intestinal,

*** p < 0.001.

## Discussion

In this study, we present real-world data on the use of methylphenidate for treating fatigue in palliative cancer care with a focus on the late palliative phase and dose response. According to our results, 46% were assessed as receiving a positive effect from the treatment and AEs were experienced by 23% and were generally mild. The most common AEs were anxiety, palpitations, and insomnia. The treatment was less likely to have an effect in patients starting treatment <4 weeks before death compared to those starting earlier in the disease trajectory.

Since previous studies have shown that CRF symptoms often increase closer to death [5], the evaluation of treatment options for CRF in late stage cancer is warranted. Our findings show that patients starting treatment in the last weeks of life were less likely to receive an effect from the treatment compared to those starting the treatment earlier in their disease trajectory. This was in contrast with our hypothesis, but an important finding for future treatment guidelines concerning methylphenidate. All functions in the body deteriorate when death approaches [31, 32], so it could be speculated that the drug-induced increase in dopamine might not have the same effect on the brain and the central nervous system at the end of life. This is strengthened by studies showing that dopamine levels decline during aging [33], although patients close to death have not been studied.

Moreover, the study shows that doses of 20 mg and above were just as well-tolerated as lower doses. The higher doses were more likely to have an effect according to the crude data but not after adjustment for confounding factors. It should be noted that the lack of significant results in the adjusted model might be due to the sample size being too small. A larger study cohort, preferably with prospectively collected data, is warranted to further study the dose-response relationship.

Only three of the seven RCTs of methylphenidate have shown a significant positive effect on fatigue [17–19]. The doses used in these three trials were 10–30 mg/day [17–19]. However, the RCTs were performed in heterogeneous cohorts having different cancer types and at different stages of the cancer disease, thus making it difficult to extrapolate to general palliative care.

The moderate effect of methylphenidate on CRF reported in the RCTs is in line with the results presented in this study, as well as the pattern of AEs. In the RCTs, insomnia was the most frequently reported AE [13–19], which is in line with our results showing that insomnia was the second most common AE.

An interesting observation is the pronounced placebo effect of psychostimulants for CRF in all the RCTs, showing similar effects in both the placebo and treatment arms [13–16]. This is further highlighted in a study by Hoenemeyer and co-workers, which compared open-label placebo to ‘usual treatment’ for the treatment of fatigue in cancer survivors [34]. Although the patients knew that they were receiving a placebo, they still experienced a significant effect from the treatment, indicating the complex etiology of fatigue.

In which patients receiving palliative care might methylphenidate have the best effect? Based on our experiences, we hypothesize that the patients suffering from an untreated /undiagnosed ADHD might receive the best effect. They may also tolerate higher doses [28, 35]. We know that methylphenidate has a positive effect on QoL and symptom burden in ADHD [26, 36, 37]. Many adults suffer from undiagnosed and untreated ADHD and when they are given methylphenidate in the palliative care setting, but with CRF as the indication, they may experience a positive effect that might not only be explained by the treatment of CRF. Patients with secondary fatigue due to, for example, anemia or malnutrition will probably experience no or very limited effect from methylphenidate. The loss of appetite and weight loss induced by methylphenidate, as well as other psychostimulants, were not recorded in the medical records. However, these are adverse effects that need to be considered when prescribing psychostimulants to patients suffering from advanced cancer.

## Limitations

This study has several limitations that should be addressed. The data are based on a review of medical records and are therefore limited to the data noted in these records. The decision to continue or terminate the treatment, and to prescribe a high or low dose, may differ between the various home care teams depending on which physician and home care team started and evaluated the treatment. Moreover, it is likely that not all AEs have been noted in the medical records, for example changes in blood pressure or weight, since these are not always monitored in patients with late stage cancer admitted to palliative care. In addition, we do not know about the patients’ compliance to the treatment. Nevertheless, since the prescribed medications are evaluated every week, and the list of medications is updated every week by the medical home care teams, medications that are not taken by the patients or those that give distressing AEs are usually terminated. The data regarding the effect of the treatment are based on the retrospective assessment by physicians reading the medical records. Although the concordance between the two independent assessors was very good, it relies solely on what is noted in the medical records. A prospective design would give much more reliable results concerning both effect and safety. Moreover, we excluded patients that were treated for less than 3 days. The results are, therefore, only applicable to patients treated with methylphenidate for 3 days or more. The study is a single-center study where methylphenidate was given in rather low doses. This is also an observational cohort study, and no power calculation was made. Instead, the study reflects the actual clinical outcome in a fixed cohort. Nevertheless, the results from this study may constitute a basis for a power calculation in a future RCT, and may help to optimize the design of such a study. Finally, previous studies have suggested that there are responders and non-responders to methylphenidate [16, 23]. ‘Non-responders’ probably terminated their treatment early and were never prescribed a higher dose. Thus, to be able to study the true optimal dose, a new RCT needs to be conducted with doses using 20 mg or higher.

Despite all these limitations, we think that this study, based on ‘real-world data’ from our clinic, might be of value to clinicians in palliative care. In RCTs, patients are generally thoroughly selected with strict inclusion and exclusion criteria, which is necessary in order to study the true effect of a treatment. However, due to selection bias in RCTs, the effect and pattern of AEs may differ from the way a treatment evolves in clinical practice. Since the present cohort includes all patients regardless of age, cancer type, or stage in their disease trajectory, that is, reflecting the true clinical situation in a palliative unit, the results are considered to be more generalizable. Since there are no guidelines concerning how late in the disease trajectory methylphenidate could be started, we think that this study may add value, showing that the treatment is less effective when started during the last weeks before death.

## Conclusion

In conclusion, methylphenidate is generally effective and well-tolerated for the treatment of CRF in patients with advanced cancer in a palliative phase. Patients with a short life expectancy of <4 weeks seem to receive less benefit from the treatment regardless of age, cancer type and dose. Doses of 20 mg and above are just as well tolerated as lower doses and should be considered to increase effectiveness. However, more research is required, preferably using prospectively collected data, before firm conclusions can be drawn.

## Data Availability

The raw data are available from the corresponding author upon reasonable request.

## References

[1] Al Maqbali M, Al Sinani M, Al Naamani Z, Al Badi K, Tanash MI. Prevalence of fatigue in patients with cancer: a systematic review and meta-analysis. J Pain Symptom Manage. 2021;61(1):167–89 e14. 10.1016/j.jpainsymman.2020.07.037 32768552

[2] Bower JE. Cancer-related fatigue – mechanisms, risk factors, and treatments. Nat Rev Clin Oncol. 2014;11(10):597–609. 10.1038/nrclinonc.2014.127 25113839 PMC4664449

[3] de Raaf PJ, de Klerk C, Timman R, Hinz A, van der Rijt CC. Differences in fatigue experiences among patients with advanced cancer, cancer survivors, and the general population. J Pain Symptom Manage. 2012;44(6):823–30. 10.1016/j.jpainsymman.2011.12.279 22795903

[4] de Raaf PJ, de Klerk C, van der Rijt CC. Elucidating the behavior of physical fatigue and mental fatigue in cancer patients: a review of the literature. Psychooncology. 2013;22(9):1919–29. 10.1002/pon.3225 23147803

[5] Hagelin CL, Wengstrom Y, Ahsberg E, Furst CJ. Fatigue dimensions in patients with advanced cancer in relation to time of survival and quality of life. Palliat Med. 2009;23(2):171–8. 10.1177/0269216308098794 18952749

[6] Klasson C, Helde Frankling M, Lundh Hagelin C, Björkhem-Bergman L. Fatigue in cancer patients in palliative care – a review on pharmacological interventions. Cancers (Basel). 2021;13(5):985. 10.3390/cancers13050985 33652866 PMC7956665

[7] Klasson C, Helde Frankling M, Warnqvist A, et al. Sex differences in the effect of vitamin D on fatigue in palliative cancer care – a post hoc analysis of the randomized, controlled trial ‘Palliative-D’. Cancers (Basel). 2022;14(3). 10.3390/cancers14030746 PMC8833647 35159013

[8] Mustian KM, Alfano CM, Heckler C, et al. Comparison of pharmaceutical, psychological, and exercise treatments for cancer-related fatigue: a meta-analysis. JAMA Oncol. 2017;3(7):961–8. 10.1001/jamaoncol.2016.6914 28253393 PMC5557289

[9] Mücke M, Cuhls H, Peuckmann-Post V, Minton O, Stone P, Radbruch L. Pharmacological treatments for fatigue associated with palliative care. Cochrane Database Syst Rev. 2015;5:CD006788. 10.1002/14651858.CD006788.pub3 PMC6483317 26026155

[10] Radbruch L, Strasser F, Elsner F, et al. Fatigue in palliative care patients – an EAPC approach. Palliat Med. 2008;22(1):13–32. 10.1177/0269216307085183 18216074

[11] Mock V, Atkinson A, Barsevick A, et al. NCCN Practice Guidelines for Cancer-Related Fatigue. Oncology (Williston Park). 2000;14(11A):151-61. 11195408

[12] Fabi A, Bhargava R, Fatigoni S, et al. Cancer-related fatigue: ESMO clinical practice guidelines for diagnosis and treatment. Ann Oncol. 2020;31(6):713–23. 10.1016/j.annonc.2020.02.016 32173483

[13] Bruera E, Yennurajalingam S, Palmer JL, et al. Methylphenidate and/or a nursing telephone intervention for fatigue in patients with advanced cancer: a randomized, placebo-controlled, phase II trial. J Clin Oncol. 2013;31(19):2421–7. 10.1200/JCO.2012.45.3696 23690414 PMC3691358

[14] Centeno C, Roji R, Portela MA, et al. Improved cancer-related fatigue in a randomised clinical trial: methylphenidate no better than placebo. BMJ Support Palliat Care. 2022;12(2):226–34. 10.1136/bmjspcare-2020-002454 33168668

[15] Escalante CP, Meyers C, Reuben JM, et al. A randomized, double-blind, 2-period, placebo-controlled crossover trial of a sustained-release methylphenidate in the treatment of fatigue in cancer patients. Cancer J. 2014;20(1):8–14. 10.1097/PPO.0000000000000018 24445757 PMC4510946

[16] Mitchell GK, Hardy JR, Nikles CJ, et al. The effect of methylphenidate on fatigue in advanced cancer: an aggregated N-of-1 trial. J Pain Symptom Manage. 2015;50(3):289–96. 10.1016/j.jpainsymman.2015.03.009 25896104

[17] Pedersen L, Lund L, Petersen MA, Sjogren P, Groenvold M. Methylphenidate as needed for fatigue in patients with advanced cancer. A prospective, double-blind, and placebo-controlled study. J Pain Symptom Manage. 2020;60(5):992–1002. 10.1016/j.jpainsymman.2020.05.023 32464260

[18] Richard PO, Fleshner NE, Bhatt JR, Hersey KM, Chahin R, Alibhai SM. Phase II, randomised, double-blind, placebo-controlled trial of methylphenidate for reduction of fatigue levels in patients with prostate cancer receiving LHRH-agonist therapy. BJU Int. 2015;116(5):744–52. 10.1111/bju.12755 24684534

[19] Roth AJ, Nelson C, Rosenfeld B, et al. Methylphenidate for fatigue in ambulatory men with prostate cancer. Cancer. 2010;116(21):5102–10. 10.1002/cncr.25424 20665492 PMC3632439

[20] Hovey E, de Souza P, Marx G, et al. Phase III, randomized, double-blind, placebo-controlled study of modafinil for fatigue in patients treated with docetaxel-based chemotherapy. Support Care Cancer. 2014;22(5):1233–42. 10.1007/s00520-013-2076-0 24337761

[21] Lee EQ, Muzikansky A, Drappatz J, et al. A randomized, placebo-controlled pilot trial of armodafinil for fatigue in patients with gliomas undergoing radiotherapy. Neuro Oncol. 2016;18(6):849–54. 10.1093/neuonc/now007 26902850 PMC4864265

[22] Spathis A, Fife K, Blackhall F, et al. Modafinil for the treatment of fatigue in lung cancer: results of a placebo-controlled, double-blind, randomized trial. J Clin Oncol. 2014;32(18):1882–8. 10.1200/JCO.2013.54.4346 24778393

[23] Auret KA, Schug SA, Bremner AP, Bulsara M. A randomized, double-blind, placebo-controlled trial assessing the impact of dexamphetamine on fatigue in patients with advanced cancer. J Pain Symptom Manage. 2009;37(4):613–21. 10.1016/j.jpainsymman.2008.03.016 18790598

[24] Homsi J, Nelson KA, Sarhill N, et al. A phase II study of methylphenidate for depression in advanced cancer. Am J Hosp Palliat Care. 2001;18(6):403–7. 10.1177/104990910101800610 11712722

[25] Bjorkhem-Bergman L, Wallin C. [Methylphenidate and cannabinoids may have a place in palliative care. Literature review and clinical experience provides evidence]. Lakartidningen. 2013;110(32–33):1409–11. 23980382

[26] Storebo OJ, Ramstad E, Krogh HB, et al. Methylphenidate for children and adolescents with attention deficit hyperactivity disorder (ADHD). Cochrane Database Syst Rev. 2015;11:CD009885. 10.1002/14651858.CD009885.pub2 PMC8763351 26599576

[27] Kapur A. Is methylphenidate beneficial and safe in pharmacological cognitive enhancement? CNS Drugs. 2020;34(10):1045–62. 10.1007/s40263-020-00758-w 32794136

[28] Kis B, Lucke C, Abdel-Hamid M, Hessmann P, Graf E, Berger M, et al. Safety profile of methylphenidate under long-term treatment in adult ADHD patients – results of the COMPAS study. Pharmacopsychiatry. 2020;53(6):263–71. 10.1055/a-1207-9851 33017854

[29] Seeman P. Schizophrenia and dopamine receptors. Eur Neuropsychopharmacol. 2013;23(9):999–1009. 10.1016/j.euroneuro.2013.06.005 23860356

[30] Sterpu B, Lindman P, Bjorkhem-Bergman L. A comparative study on decision and documentation of refraining from resuscitation in two medical home care units in Sweden. BMC Palliat Care. 2019;18(1):80. 10.1186/s12904-019-0472-z 31623585 PMC6798351

[31] Flatt T. A new definition of aging? Front Genet. 2012;3:148. 10.3389/fgene.2012.00148 22936945 PMC3425790

[32] Lunney JR, Lynn J, Foley DJ, Lipson S, Guralnik JM. Patterns of functional decline at the end of life. JAMA. 2003;289(18):2387–92. 10.1001/jama.289.18.2387 12746362

[33] Mukherjee J, Christian BT, Dunigan KA, et al. Brain imaging of 18F-fallypride in normal volunteers: blood analysis, distribution, test-retest studies, and preliminary assessment of sensitivity to aging effects on dopamine D-2/D-3 receptors. Synapse. 2002;46(3):170–88. 10.1002/syn.10128 12325044

[34] Hoenemeyer TW, Kaptchuk TJ, Mehta TS, Fontaine KR. Open-label placebo treatment for cancer-related fatigue: a randomized-controlled clinical trial. Sci Rep. 2018;8(1):2784. 10.1038/s41598-018-20993-y 29426869 PMC5807541

[35] Ching C, Eslick GD, Poulton AS. Evaluation of methylphenidate safety and maximum-dose titration rationale in attention-deficit/hyperactivity disorder: a meta-analysis. JAMA Pediatr. 2019;173(7):630–9. 10.1001/jamapediatrics.2019.0905 31135892 PMC6547117

[36] Mattos P, Louza MR, Palmini AL, de Oliveira IR, Rocha FL. A multicenter, open-label trial to evaluate the quality of life in adults with ADHD treated with long-acting methylphenidate (OROS MPH): Concerta Quality of Life (CONQoL) study. J Atten Disord. 2013;17(5):444–8. 10.1177/1087054711434772 22334621

[37] Tripp G, Wickens JR. Neurobiology of ADHD. Neuropharmacology. 2009;57(7–8):579–89. 10.1016/j.neuropharm.2009.07.026 19627998

